# Though not Reservoirs, Dogs might Transmit *Leptospira* in New Caledonia

**DOI:** 10.3390/ijerph110404316

**Published:** 2014-04-17

**Authors:** Noellie Gay, Marie-Estelle Soupé-Gilbert, Cyrille Goarant

**Affiliations:** Institut Pasteur in New Caledonia, 9-11 avenue Paul Doumer, BP 61 98845 NOUMEA Cedex, New Caledonia; E-Mails: ngay@pasteur.nc (N.G.); msoupe@pasteur.nc (M.-E.S.-G.)

**Keywords:** *Leptospira*, epidemiology, mammal, dog, reservoir, vector

## Abstract

*Leptospira* has been a major public health concern in New Caledonia for decades. However, few multidisciplinary studies addressing the zoonotic pattern of this disease were conducted so far. Here, pig, deer and dog samples were collected. Analyses were performed using molecular detection and genotyping. Serological analyses were also performed for dogs. Our results suggest that deer are a reservoir of *L. borgpetersenii* Hardjobovis and pigs a reservoir of *L. interrogans* Pomona. Interestingly, 4.4% of dogs were renal carriers of *Leptospira*. In dog populations, MAT results confirmed the circulation of the same *Leptospira* serogroups involved in human cases. Even if not reservoirs, dogs might be of significance in human contamination by making an epidemiological link between wild or feral reservoirs and humans. Dogs could bring pathogens back home, shedding *Leptospira* via their urine and in turn increasing the risk of human contamination. We propose to consider dog as a vector, particularly in rural areas where seroprevalence is significantly higher than urban areas. Our results highlight the importance of animal health in improving leptospirosis prevention in a One Health approach.

## 1. Introduction

Leptospirosis is a widespread zoonosis in the tropics and notably the Pacific Islands [1,2]. It has been a major public health concern in New Caledonia (NC) for decades and was notably classified as a notifiable disease in 1991. Humans get infected when abraded skin or mucous membranes come into contact with contaminated kidneys, urine or urine-contaminated environments [3].

*Leptospira* strains are maintained in different animal species and excreted in the urine of asymptomatic chronically infected individuals [4,5]. Virtually any mammal species can act as a reservoir, characterized by a sustained non-symptomatic renal carriage [6] of a co-adapted *Leptospira* strain [4]. When not co-adapted, *Leptospira* do not chronically colonize kidneys of mammals, then considered as accidental hosts and frequently showing clinical signs when infected. At a population scale, a low prevalence of renal carriage (around or below 1%) is expected in accidental hosts whereas it can reach more than 10% in reservoir populations [7]. Exposure to various mammals was found to be a risk for human leptospirosis in NC [8], namely rodents, horses, cattle and pigs and the role of indirect contamination via environmental exposure was highlighted [9].

Few mammals are present in NC: nine bat species, all indigenous, four introduced rodents (*Rattus exulans*, *Rattus rattus*, *Rattus norvegicus* and *Mus musculus*) and some introduced domestic mammals such as dog, cat, cattle, horse, goat and Rusa deer (*Rusa timorensis*) [10]. Two *Leptospira* species circulate in NC: *L. interrogans* and *L. borgpetersenii* [11] including five and two genotypes, respectively [12]. These are *L. interrogans* serogroups (sg) Icterohaemorrhagiae (accounting for *ca.* 50%–60% of human cases yearly), Pomona (*ca.* 5%), Pyrogenes (15%–25%), Australis (5%–10%), Bataviae (<5%) and *L. borgpetersenii* sg Ballum (*ca.* 10%) and serovar Hardjobovis (never evidenced in human cases) [12]). Thus, paralleling its limited mammal diversity, NC also presents a low diversity of pathogenic *Leptospira* compared to inland countries or its neighbor Australia (http://www.health.gov.au/internet/main/publishing.nsf/Content/cda-phlncd-leptospirosis.htm). Despite extensive surveillance for more than two decades and serological surveys using the Microscopic Agglutination Test [9], some strains otherwise widely distributed were never evidenced in NC. Of note, serogroup Canicola, which reservoir is dog worldwide [13] was never evidenced in NC.

Rodents are recognized as the most significant reservoir of leptospires worldwide [3,5,14]. The overall prevalence of *Leptospira* spp. in rodents from NC was 26.7% [15]. Higher rodent abundance and *Leptospira* prevalence were evidenced during hot rainy periods. No difference between species was found, however, commensal species (*R. norvegicus* and *M. musculus*) had a higher prevalence than sylvatic rodents [15]. Mice maintain *L. borgpetersenii* sg Ballum and Norway rats are the reservoir of *L. interrogans* sg Icterohaemorrhagiae. Laboratory diagnoses of human cases are performed at the reference laboratory, Institut Pasteur de Nouvelle-Calédonie, which provides biological data to the Health authority for epidemiological surveillance purpose. Surveillance data show that leptospires involved in the majority of human cases in NC are maintained by rodents (Icterohaemorrhagiae in the three rat species and Ballum in the mice and some black rats [15]), but that three other genotypes, corresponding to serogroups Pomona, Pyrogenes and Australis, were also involved in a significant number of human cases [12].

The mammal reservoirs of these latter *Leptospira* are currently investigated using molecular approaches similar to the ones used for characterizing human cases [12,16] and the rodent reservoir [15]. Thus, a field-to-laboratory survey was set up to update data on pathogenic *Leptospira* carriage by animals in NC. To achieve this goal, we estimated the prevalence of renal infection by *Leptospira* in deer, pigs and dogs and genotyped the strains evidenced in these animals. 

## 2. Experimental Section

From March to October 2013, a total of 519 samples were collected for molecular analysis. Pig and deer kidneys were sampled at slaughterhouses in Paita and Bourail, respectively. Samples from feral pigs and deer were collected by the Conservatoire des Espaces Naturels responsible for the environmental management of invasive species in NC. Eighty two dog kidney samples were obtained from the pound of Nouméa (urban dogs), 13 dog urine specimens were from apparently healthy dogs sampled in various tribes (one from Poindimié and 12 from Houailou). 

Animals from slaughterhouses were considered as clinically healthy when sampled because pre-slaughter veterinary controls systematically apply in slaughterhouses. Pound-euthanized dogs were all stray dogs, but also apparently healthy. They had been kept captive for at least eight days in the pound, where rodent control is regularly implemented. Therefore, if dogs in the pound were infected by *Leptospira*, the contamination was considered to be acquired before capture. Urine samples were collected from live apparently healthy animals, buffered with 10% 10X phosphate buffer saline and stored in a cool box until transfer to the laboratory for direct DNA extraction. Kidney samples were immediately placed and stored in 95% alcohol for postponed DNA extraction as described before [15]. A single sample from one kidney was taken from each individual animal and was considered as representative of the animal kidneys.

Dog venous blood was collected immediately after death for serology from 31 urban dogs at the pound of Nouméa in 2010–2011 and from 47 live rural dogs in 2013 (mostly from tribes in the Northern Province in Hienghène, Houailou and Ouégoa).

### 2.1. DNA Extraction

A small piece of kidney tissue (*ca.* 25 mg) was dissected and rehydrated overnight in 1,000 μL sterile water at 4 °C. Water was then removed; 50 μL of 1X sterile phosphate buffer saline was added to the kidney sample before DNA extraction using the QIAamp DNA mini kit (QIAGEN, Auckland, New Zealand) following manufacturer’s instructions for tissue. Urine samples were extracted following manufacturer’s instructions. All DNA concentrations were standardized to 50 ng/μL after measurement of the concentration with a NanoDrop 2000 (Thermo Scientific, Scoresby, Victoria, Australia).

### 2.2. Molecular Tools

*Leptospira* was detected using two different real time PCRs, both targeting *lipL32* [17,18]. If the sample tested positive, the *lfb1* gene was amplified using SYBR Green technology [19]. To check for the absence of inhibitors that could lead to false negative results, every negative DNA sample was amplified with a universal 16S rDNA PCR. The ones containing inhibitors were repeatedly extracted and submitted to amplification. If the second DNA extract also contained inhibitors, the sample was not considered in the analysis.

### 2.3. Leptospira Genotyping by Sequencing

*The lfb1* amplification products from positive samples were purified using the MinElute PCR purification kit (QIAGEN) and directly sequenced for genotyping as described by Perez *et al.* [12].

### 2.4. Serology

Microscopic Agglutination Tests (MAT) were used to check 78 dog sera for *Leptospira*-specific antibodies with the local panel used for human diagnosis [8] using a 1:100 positivity threshold. This MAT panel was developed and optimized for leptospirosis diagnosis in New Caledonia and is described in Table 1. 

**Table 1:** New Caledonian panel of *Leptospira* strains used for the MAT.

Species	Serogroup	serovar	Strain
*L. interrogans*	Australis	Australis	Ballico
*L. interrogans*	Autumnalis	Autumnalis	Akiyami A
*L. borgpetersenii*	Ballum	castellonis	Castellon 3
*L. interrogans*	Bataviae	Bataviae	Van Tienen
*L. interrogans*	Canicola	Canicola	Hond Utrecht
*L. interrogans*	Icterohaemorrhagiae	Icterohaemorrhagiae	Verdun
*L. interrogans*	Icterohaemorrhagiae	Copenhagenii	Winjberg
*L. noguchi*	Panama	Panama	CZ 214 K
*L. interrogans*	Pomona	Pomona	Pomona
*L. interrogans*	Pyrogenes	Pyrogenes	Salinem
*L. borgpetersenii*	Tarassovi	Tarassovi	Mitis Johnson
*L. biflexa*	Semarranga	Patoc	Patoc I

Following standard recommendations, the infecting serogroup was designated as the serogroup of the strain with a titer at least 4-fold the titer of the other strains. When this was not possible (co-agglutinations or highest titers with the non-pathogenic *L. biflexa* Patoc I), the MAT was considered as positive for an unidentified serogroup. Statistical analyses were computed with R software and Fisher's exact tests were used.

## 3. Results

### 3.1. Leptospira Carriage

From qPCR amplification, 14 kidney DNA extracts demonstrated PCR inhibitors and were not integrated in statistical analysis (nine deer, four dogs, one pig).

Pathogenic *Leptospira* renal carriage was detected in all three species investigated. The detailed results are shown in Table 2. The prevalence was highly variable depending on the species, being as high as 13% in deer, reaching 8.7% in pigs and 4.4% in dogs.

**Table 2:** Distribution of *Leptospira* among mammals sampled

Mammal	Source	Sample size	PCR inhibition	Positive	Negative	Prevalence	95% CI
Deer *	Hunting	85	9	25	167	13.02	[8.26–17.78]
	Slaughtered	107					
Pig *	Feral	94	0	6	88	6.38	[1.44–11.32]
	Farmed	138	1	14	123	10.22	[5.15–15.29]
Dog	Urban pound (kidney)	82	4	3	75	4.4	[0.19–8.61]
	Tribes (urine)	13	0	1	12		

* Because deer are captured from feral populations and only ranched for a few days or weeks before slaughter, hunted or slaughtered deer should not be considered as distinct populations. Oppositely, feral and farmed pigs are distinct populations, providing the opportunity to evaluate the prevalence in both populations.

### 3.2. Strain Identification by Genotyping

**Figure 1:**
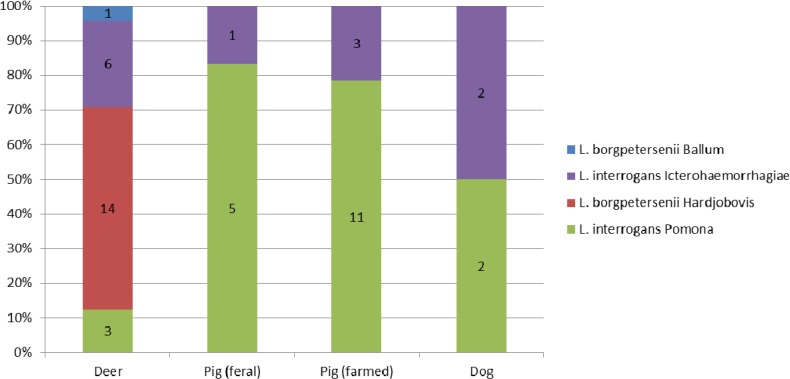
*Leptospira* identification among positive Mammals.

The molecular identification of strains provided information about the infecting *Leptospira* at the species and serogroup level (Figure 1). The majority of *Leptospira* evidenced in deer were *L. borgpetersenii*, the DNA sequence pointing to Hardjobovis and positive pigs were mostly carriers of *L. interrogans*, the sequence pointing to sg Pomona. In dogs, both *L. interrogans* sg Pomona (one rural dog and one urban dog) and *L. interrogans* sg Icterohaemorrhagiae (two urban dogs) were evidenced.

### 3.3. MAT on Dog Sera

Overall 24 (30.8%) of the 78 dog sera tested were found seropositive with MAT. From 31 urban dogs from the pound, four were reactive with sg Icterohaemorrhagiae (Table 3). The majority of sera from rural dogs were seropositive for sg Australis and Icterohaemorragiae (Table 3). Seroprevalence was significantly higher for rural dogs (*p* < 0.005).

**Table 3:** Putative serogroup in MAT-positive dogs according to their origin.

Putative Serogroup	Australis	Icterohaemorrhagiae	Canicola	Pyrogenes	Unknown *
Urban dogs (*n* = 31)	0	4	0	0	1
Rural dogs (*n* = 47)	3	11	0	1	4

* co-agglutinations or highest titre for non-pathogenic *L. biflexa* Patoc.

## 4. Discussion

In our study, deer were identified as a reservoir of *L. borgpetersenii* Hardjobovis as suggested by Perez *et al.* [12] in a former study. Interestingly, despite frequent interactions between deer and hunters, *L. borgpetersenii* Hardjobovis has never been identified in human cases in New Caledonia [12]. Whether it is exclusively transmitted between animals or if it induces only few symptoms in humans, resulting in an underreporting, remains to be determined. Additionally, deer were probably involved in the circulation of Pomona in NC, though more investigations are needed to better understand the role of deer in Pomona maintenance and circulation.

Among *Leptospira* positive pigs, most carried *L. interrogans* sg Pomona, whether farmed or feral. Thus, pigs might be a reservoir of this serogroup in NC as reported in New Zealand [20]. Interestingly, in 2013, 8% of human leptospirosis cases in NC involved *L. interrogans* sg Pomona (our unpublished data). Consequently, prevention measures should be encouraged to limit contacts of humans with pig manure or with kidneys and urine at slaughter. Our results also suggest that both deer and pigs (feral or farmed) are exposed to rodent-borne leptospirosis, as evidenced by some carriage of Icterohaemorrhagiae or Ballum, maintained by rats and mice [15].

In our study, *Leptospira* renal carriage prevalence in dogs was 4.4%, molecular identification of the infecting strains pointing to *L. interrogans* sg Pomona and Icterohaemorrhagiae. This survey again failed to evidence any Canicola, further suggesting its absence in New Caledonia. And yet, it is usually considered that dogs are susceptible to Pomona and Icterohaemorrhagiae rather than possible reservoirs [13,21], though possible chronic carriage of Pomona in some dogs is still a matter of debate. However, in our study, the animals surveyed displayed no clinical symptoms when sampled. The three dogs from the pound were active and apparently healthy for at least 8 days as they were caught wandering at least one week before euthanasia. These dogs were most probably at a late stage of a non-lethal leptospirosis, currently recovering but still carrying leptospires in their kidneys and shedding it in their urine. It is recognized that *Leptospira* excretion in urine might be intermittent. As a result, the detection of carrier animals using urine specimens might be less a sensitive technique when compared with detection from kidney tissue. Because rural dogs had to be sampled live, we could only obtain urine specimens, possibly under-regarding the prevalence in this particular canine population.

This survey confirms that dogs are most probably not a reservoir host for *Leptospira* in NC as the prevalence was low and serogroup Canicola was not identified in any specimen. However, a significant proportion of dogs were carrying pathogenic leptospires in their kidneys. We hypothesise that at a population scale, though not maintenance hosts, dogs are probably involved in the circulation of pathogenic *Leptospira*. Our results suggest that these dogs were infected after direct or indirect contact with pig, deer or rats, or with another infected dog. Such dogs might be regarded as “vectors”: this mammal, living in close contact with humans, is exposed to various sources of direct or environmental contamination and is more likely to interact with both other mammal reservoirs (hunting, wandering) and humans. Dogs are highly exposed to zoonotic and environmental contamination and could bring pathogens back home, shedding *Leptospira* via their urine in the household environment and in turn increasing the risk of human contamination. Further supporting our hypothesis, MAT results confirmed the circulation in dog populations of the same *Leptospira* strains involved in human cases (sg Icterohaemorrhagiae, Australis and Pyrogenes being the most prevalent) [12]. This particular role for dogs was already suggested in other places. In the island of Barbados, where Canicola is also absent, epidemiological data using serology suggests a similar role for dogs in transmitting leptospirosis from a wild and environmental reservoir to humans [22]. An increase in human leptospirosis in Germany was linked to a resurgence of canine leptospirosis [23]. In Nicaragua, patients were significantly more likely than controls to own seropositive dogs [24,25], again suggesting transmission between dogs and their owners or contamination from a common source. Our study, by evidencing renal carriage of pathogenic *Leptospira* using molecular detection and typing, strongly supports this role of dogs as an epidemiological link between the environment, wild fauna and humans.

Rural environments are thought to pose a higher risk of infection for dogs because of more frequent interactions with wildlife and watered environments [26]. Our molecular survey mostly included urban dogs (86%) and cannot statistically address this specific issue. However, our serological results confirm a significantly higher seroprevalence in rural (40.4%) than in urban (16.1%) dogs (*p* < 0.005), reinforcing the hypothesis that rural dogs could be a risk for human leptospirosis in NC. Improving the awareness of dog owners and the prevention of canine leptospirosis, particularly in rural places, could be a valuable asset for human leptospirosis prevention.

Leptospirosis infection in dogs can be treated with appropriate antibiotics, which are effective in preventing urinary shedding [27]. The control of dog populations using castration might also prove useful in reducing *Leptospira* circulation, as suggested by Yoak and collaborators [28]. Lastly, it has been shown that vaccination could induce a protection against both clinical leptospirosis and renal carriage and shedding [29]. As observed in our survey in NC, the serovar Pomona was increasingly identified in dogs in North America [26,30] and Europe [31] leading to the inclusion of the Pomona valence in a new dog vaccine in North America [32]. If effective in preventing renal carriage and urinary shedding, this new vaccine could be used in New Caledonian dogs and could improve prevention for both dogs and owners. Consequently, the role of animal health in public health needs to be considered in a One Health approach. As suggested before [33], the implementation of a surveillance system for canine leptospirosis, using dogs as sentinels for human risk assessment, could also provide a valuable tool for estimating and in turn minimizing the risk for humans. 

## 5. Conclusions

Our results suggest that dogs are not a *Leptospira* reservoir host and further failed to identify Canicola (using both serological and molecular tools), a serogroup most probably absent in NC. However, dogs may, during recovery from mild *Leptospira* infections, contribute to human leptospirosis by bringing pathogenic strains closer to humans and their households. We propose to consider dogs as vectors, ensuring the link from primary animal reservoirs to humans through their role as companion animals. The contribution of dogs, particularly in rural settings, as a vector between wild or feral reservoirs and human should be further investigated.
